# Receptor activity‐modifying protein 1 regulates mouse skin fibroblast proliferation via the Gαi3-PKA-CREB-YAP axis

**DOI:** 10.1186/s12964-022-00852-0

**Published:** 2022-04-12

**Authors:** Siyuan Yin, Ru Song, Jiaxu Ma, Chunyan Liu, Zhenjie Wu, Guoqi Cao, Jian Liu, Guang Zhang, Huayu Zhang, Rui Sun, Aoyu Chen, Yibing Wang

**Affiliations:** 1 Department of Plastic Surgery, Shandong Provincial Qianfoshan Hospital, Cheeloo College of Medicine, Shandong University, Jinan, 250012 Shandong People’s Republic of China; 2 Jinan Clinical Research Center for Tissue Engineering Skin Regeneration and Wound Repair, The First Affiliated Hospital of Shandong First Medical University & Shandong Provincial Qianfoshan Hospital, Jinan, 250014 Shandong People’s Republic of China; 3 Department of Plastic Surgery, The First Affiliated Hospital of Shandong First Medical University & Shandong Provincial Qianfoshan Hospital, Jinan, 250014 Shandong People’s Republic of China

**Keywords:** RAMP1, Fibroblast proliferation, PKA, CREB, YAP, Transcription factor, Wound healing

## Abstract

**Background:**

Skin innervation is crucial for normal wound healing. However, the relationship between nerve receptors and wound healing and the intrinsic mechanism remains to be further identified. In this study, we investigated the role of a calcitonin gene-related peptide (CGRP) receptor component, receptor activity‐modifying protein 1 (RAMP1), in mouse skin fibroblast (MSF) proliferation.

**Methods:**

In vivo, Western blotting and immunohistochemical (IHC) staining of mouse skin wounds tissue was used to detect changes in RAMP1 expression. In vitro, RAMP1 was overexpressed in MSF cell lines by infection with Tet-On-Flag-RAMP1 lentivirus and doxycycline (DOX) induction. An IncuCyte S3 Live-Cell Analysis System was used to assess and compare the proliferation rate differences between different treatment groups. Total protein and subcellular extraction Western blot analysis, quantitative real-time-polymerase chain reaction (qPCR) analysis, and immunofluorescence (IF) staining analysis were conducted to detect signalling molecule expression and/or distribution. The CUT & RUN assay and dual-luciferase reporter assay were applied to measure protein-DNA interactions.

**Results:**

RAMP1 expression levels were altered during skin wound healing in mice. RAMP1 overexpression promoted MSF proliferation. Mechanistically, total Yes-associated protein (YAP) and nuclear YAP protein expression was increased in RAMP1-overexpressing MSFs. RAMP1 overexpression increased inhibitory guanine nucleotide-binding protein (G protein) α subunit 3 (Gαi3) expression and activated downstream protein kinase A (PKA), and both elevated the expression of cyclic adenosine monophosphate (cAMP) response element-binding protein (CREB) and activated it, promoting the transcription of *YAP*, elevating the total YAP level and promoting MSF proliferation.

**Conclusions:**

Based on these data, we report, for the first time, that changes in the total RAMP1 levels during wound healing and RAMP1 overexpression alone can promote MSF proliferation via the Gαi3-PKA-CREB-YAP axis, a finding critical for understanding RAMP1 function, suggesting that this pathway is an attractive and accurate nerve target for skin wound treatment.

**Video Abstract**

**Supplementary Information:**

The online version contains supplementary material available at 10.1186/s12964-022-00852-0.

## Background

Based on its surface area, the skin is the largest organ in humans [1–3], and skin wound healing is a dynamic and highly regulated and coordinated process involving haemostasis, inflammation, angiogenesis, growth, re-epithelialization and remodelling [4]. In this process, fibroblasts play a crucial role from the late inflammatory phase to final epithelization through secretion, migration, proliferation and transdifferentiation [3, 5].

Nerves and neuromodulators, including calcitonin gene-related peptide (CGRP), play essential roles in different steps of the skin wound healing process [6, 7] by targeting all skin tissues and cells, including keratinocytes, fibroblasts, vascular endothelial cells and immune cells [7–11]. Damaged or abnormal nerves and/or nerve function can result in chronic wounds [12]; additionally, neurogenic stimuli can promote the repair not only of denervation-related wounds but also of ulcers, ischaemic wounds and other chronic wounds [13–15], among which CGRP, a 37-amino acid neuropeptide, is an attractive candidate [16]. However, the short half-life of CGRP in plasma limits its long-term application [17]; therefore, we focused on its receptor, which is a heterodimer comprising three subunits: a seven-transmembrane guanine nucleotide-binding protein (G protein)-calcitonin receptor-like receptor (CLR), receptor activity-modifying protein 1 (RAMP1) and receptor component protein (RCP) [16, 18]. RAMP1 is a small protein with a single transmembrane domain that, in addition to endowing CGRP with binding specificity, regulates receptor trafficking, signalling and cell proliferation, migration and differentiation [19]. RAMP1 is highly expressed in skin cells and is related to wound healing. Chie et al. [20] reported that RAMP1 plays a critical role in wound healing by promoting angiogenesis and lymphangiogenesis. Additionally, Toshiaki et al. [21] reported that RAMP1 can stimulate lymphangiogenesis and restore lymphatic flow to improve lymphedema. However, no direct study on RAMP1 and skin fibroblasts has been performed.

Yes-associated protein (YAP) is a downstream effector of the Hippo pathway and plays an essential role in the control of cell proliferation and survival. YAP is also important for wound healing and is highly expressed in fibroblasts inside and outside the wound bed in the early wound healing phase [22–24]. G protein-coupled receptor (GPCR)-mediated signalling can modulate YAP/TAZ activity either positively or negatively, depending on the different cell types and various signals, receptors, and adaptor proteins involved [25]. As a class-B family [26] GPCR protein, CGRP, in conjunction with RAMP1, can stimulate YAP/TAZ expression and activity to promote liver regeneration [27]. The classical pathway of GPCRs involves stimulatory Gα subunit (Gαs) proteins simulating adenylyl cyclase (AC) to produce cyclic adenosine monophosphate (cAMP) and elicit protein kinase A (PKA) and downstream cAMP response element-binding protein (CREB), while the inhibitory Gα subunit (Gαi) protein inhibits this process [28]. However, the Gi protein can stimulate AC2 to activate PKA and its downstream signalling cascade [29]. Yu et al. [30] reported that PKA can phosphorylate YAP and modulate cell proliferation and differentiation. Similarly, using inducible PKA transgenic mice, Zhang et al. [31] found that CLR can activate PKA and prevent YAP activation and nuclear translocation. Wang [32] discovered that CREB interacts with YAP to promote liver cancer tumorigenesis. Considering these previous studies, we aimed to demonstrate the function of RAMP1 in skin fibroblasts, determine whether YAP is the main effector of RAMP1, and then identify the downstream pathway.

In the current study, we proved that RAMP1 expression levels are altered during skin wound healing in mice, constructed a RAMP1-overexpressing mouse skin fibroblast (MSF) cell line and found that RAMP1 promoted the proliferation of these MSFs through the Gαi3-PKA-CREB-YAP axis. This study provides new insights into the effects of RAMP1 on fibroblasts and some hints to more accurately regulate nerve innervation during wound healing.

## Methods and materials

### Cell culture and reagents

The MSF cell line (Guangzhou Jennio Biotech Co., Ltd., Guangzhou, China) was grown in high glucose (4.5 g/L glucose) Dulbecco’s modified Eagle’s medium (DMEM; Gibco, Thermo Fisher Scientific, MA, USA) with 10% tetracycline-free foetal bovine serum (FBS; Gibco) and maintained at 37 ℃ in a humidified 5% CO_2_ incubator. The cells were treated with H-89 dihydrochloride (1 μM; CAS. 130964-39-5; MedChemExpress, LLC, USA), bucladesine sodium (BUC; 0.01 μM; CAS. 16980-89-5; MedChemExpress), verteporfin (VP; 0.01 μM; CAS. 129497-78-5; MedChemExpress), KG-501 (10 μM; CAS. 18228-17-6; MedChemExpress) or the relative concentration of solvent dimethyl sulfoxide (DMSO; CAS. 67-68-5; MP Biomedicals, LLC, USA).

### Doxycycline (DOX)-inducible lentivirus infection and small interfering RNA transfection

MSFs were infected with Tet-On-Flag-RAMP1 lentivirus or Tet-On-Flag-vector lentivirus (GeneChem Co., Ltd., Shanghai, China) for 24 h (the primer sequences used are shown in Additional file 1: Table S1), and then the cells were selected with growth medium containing puromycin (3 μg/ml; CAS. 58-58-2; Beijing Solarbio Science & Technology Co., Ltd., Beijing, China) for 48 h to obtain stable and highly pure clones. These clones were then treated with DOX (5 μg/ml; CAS. 564-25-0; MedChemExpress) to induce the expression of Flag-RAMP1 or its control form. RAMP1 overexpression MSFs were reverse-transfected with 50 nM small interfering RNAs (siRNAs) specifically targeting Gαi3 (siGαi3-1 and siGαi3-2; Guangzhou RiboBio Co., Ltd., Guangzhou, China) or a negative control sequence (siNC; RiboBio) using RiboBio transfection reagent. After 48 h, the cells were harvested for RNA and protein extraction and detection. The gene sequences used for the Gαi3 siRNA and its negative control are provided in Additional file 1: Table S2.

### Cell proliferation assay

MSFs with different treatments were seeded in a 96-well plate (Corning Incorporated, USA) at an initial 3000 cells per well, and then the plate was placed in an IncuCyte S3 Live-Cell Analysis System, where real-time images were captured every 6 h for 72 h or 2000 cells per well were imaged for 96 h. Photographs of the cells were taken from two separate regions of each well using a 10× objective. Values from the two regions of each well were pooled and averaged across three replicates, and the proliferation rate was determined as the area confluence ratio normalized to hour 0 using IncuCyte S3 software (Essen BioScience, Ann Arbor, MI, USA).

### Animals

C57BL/6J male mice (8–10 weeks old; weight 30–35 g) (Beijing Vital River Laboratory Animal Technology Co., Ltd, Beijing, China) were used as a skin wound model in this study. All the animals were housed individually under a 12:12 h light/dark cycle at a controlled constant temperature (25 ± 1 °C) and humidity (60 ± 5%). All the experiments were approved by the ethics committee of The First Affiliated Hospital of Shandong First Medical University & Shandong Provincial Qianfoshan Hospital (Approval Number: SYDWLS[2021]002) and were performed in accordance with the guidelines and regulations.

### Skin wound model and evaluation of wound healing

After anaesthesia with 1% pentobarbital sodium, the mouse was depilated and disinfected. On both side of the dorsal part, approximately 7 mm away from midline, 2 points 4 cm away from the base of the neck were marked as the central points of the wound preparation. Next, a sterile, disposable 5 mm biopsy punch tool was used to create a circular wound outline at this point, along with a pair of iris scissors (with curved tips) to excise the circular piece of tissue to make a full-thickness wound. The time of wound induction was defined as hour 0. After wound preparation was completed, the mice were housed individually (one mouse per cage).

Twenty male mice were randomly divided into five groups: (1) siGαi3-2 group (treated with 5 nmol); (2) VP group (treated with 100 nmol VP); (3) H-89 group (treated with 100 nmol H-89); (4) BUC group (treated with 100 nmol BUC); and (5) KG-501 group (treated with 100 nmol KG-501). 24 h (days 1) after wound induction, the diluent was subcutaneously administered at the edge of the wound of the right side for 7 consecutive days, while the wound of the left side was administered siNC (5 nmol) or DMSO at a coordinated dose (10 μl) as a self-control. At hours 0 (days 0), 24 h (days 1) and days 3, 5 and 7 postinjury, a ruler was placed below the wound, and the wound was photographed at a fixed height for wound healing evaluation. The wound healing rate was calculated as a percentage of the initial wound area by defining the initial wound area as 0% closure using ImageJ software (U.S. National Institutes of Health, Bethesda, MD, USA).

### Skin wound tissue sample process

A total of 12 male C57BL/6J mice (8–10 weeks old; weight 30–35 g) were used for skin wound tissue sample process. At 24 h (days 1) and days 3, 5 and 7 after wound excision, three randomly selected mice were anaesthetized, and the periwound skin was lifted with forceps. The wound tissue, including an additional 3 mm of normal skin around the wound, was harvested with a scalpel and divided into two parts along with the central axis.

Half of the above tissue was divided into two parts and rapidly frozen in a 1.5 ml snap cap tube in liquid nitrogen and stored at − 80 °C for subsequent molecular analysis. Wound tissue protein was extracted using a Minute™ Total Protein Extraction Kit for Skin Tissue (#SA-01-SK, Invent Biotechnologies, Inc., USA).

The other half of the above tissue was fixed in 10% formalin solution, embedded in paraffin and sectioned at 3 μm for histochemical analyses which were performed by haematoxylin–eosin (H&E) staining and immunohistochemical (IHC) staining. Deparaffinization, rehydration, antigen retrieval, endogenous peroxidase blocking, and goat serum (#SP-9001; ZSGB-BIO, Beijing, China) blocking of paraffin sections were performed as previously described [33–35]. Next, the wound skin tissue sections were incubated with primary antibody against RAMP1 (#ab156575; Abcam Plc, Cambridge, UK) at a dilution of 1:200 at 4 °C overnight. On the second day, all sections were incubated with biotin-labelled goat anti-rabbit IgG polymer for 15 min at room temperature and incubated with horseradish enzyme-labelled streptavidin working solution for 15 min at room temperature. Finally, the slides were counterstained with diaminobenzidine (DAB; #ZLI-9018, ZSGB-BIO) and haematoxylin (CAS. 517-28-2; Beijing Solarbio Science & Technology). For H&E staining, after deparaffinization and rehydration, slides were stained with haematoxylin and eosin (CAS. 17372-87-1; Beijing Solarbio Science & Technology) according to the manufacturer’s instructions. All the sections were then dehydrated, cleared, and sealed. The images were observed and captured using an Olympus IX73 microscope (Olympus, Tokyo, Japan). The mean density was calculated as the integrated optical density (IOD)/area using ImageJ software.

### Subcellular fractionation

To separate cytosolic and nuclear fractions, a Minute^™^ cytoplasmic and nuclear extraction kit (#SC-003; Invent Biotechnologies) was used according to the manufacturer’s protocol. Briefly, cells were washed in cold phosphate-buffered saline (PBS; #B310KJ, Shanghai BasalMedia Technologies Co., Ltd., Shanghai, China) and lysed following incubation with cytoplasmic extraction buffer on ice for 5 min with vigorous vortexing for 15 s. Next, the lysates were centrifuged at 14,000 g for 5 min at 4 ℃ to obtain the cytosolic and membrane fractions (supernatant) and nuclear fraction (pellet). The nuclear pellet was then washed with PBS three times, lysed with nuclear extraction buffer while undergoing vigorous vertexing and centrifuged in a prechilled filter cartridge with a collection tube to obtain the nuclear extract.

### Western blot analysis

Western blot analysis was performed as previously described [36]. The following antibodies and reagents used: primary antibodies against Flag (#14793), PCNA (#13110), PKA C-α (#4782), CREB (#9197), pCREB (S133) (#9198), YAP (#14074) and pYAP (S127) (#13008) were purchased from Cell Signaling Technology, Inc. (diluted 1:1000; CST, MA, USA), a primary antibody against RAMP1 (#ab156575) was purchased from Abcam Plc (diluted 1:1000; Cambridge, UK), and a primary antibody against Gαi3 (#sc-365422) was purchased from Santa Cruz Biotechnology, Inc. (diluted 1:500; TX, USA); horseradish peroxidase (HRP)-linked anti-rabbit IgG (#7074) and HRP-linked anti-mouse IgG (#7076) secondary antibodies were purchased from CST (diluted 1:5000); and total protein-labelling No-Stain^™^ reagent (#A44449) was purchased from Thermo Fisher Scientific.

### Quantitative real-time polymerase chain reaction (qPCR) analysis

RNA isolation and reverse transcription were performed according to our previously reported method [36]. Quantitative analysis was performed using a SYBR^®^ Green Premix *Pro Taq* HS qPCR kit (#AG11701; Accurate Biotechnology, Hunan, China) and the QuantSudio^™^ 3 real-time PCR system (Applied Biosystems, Thermo Fisher Scientific) according to the manufacturer’s instructions. β-Actin mRNA was used as an internal control, and the primers used are shown in Additional file 1: Table S3.

### Immunofluorescence (IF) staining

MSFs subjected to different treatments were washed with PBS, fixed with 4% paraformaldehyde (#P0099; Beyotime Institute of Biotechnology, Jiangsu, China) at room temperature for 10 min and permeabilized with immunostaining permeabilization buffer (#P0097; Beyotime) for 10 min. Next, slides with cells were incubated with primary antibody at 4 °C overnight and then with secondary antibody anti-rat Alexa Fluor-488 (#4416; diluted 1:200; CST), anti-rat Alexa Flour-555 (#4417; diluted 1:200; CST), anti-rabbit Alexa Fluor-488 (#4412; diluted 1:200; CST) or anti-rabbit Alexa Fluor-555 (#4413; diluted 1:200; CST) at room temperature for 1 h. Nuclei were stained with DAPI (#62248; Thermo Fisher Scientific) at room temperature for 30 min. Finally, the slides were covered with ProLong Gold antifade reagent (#P36934; Invitrogen), observed and recorded using a Nikon Eclipse Ti2 confocal microscope (Nikon Instruments (Shanghai) Co., Ltd., Shanghai, China). The following primary antibodies were used: rabbit anti-Flag (#14793), anti-PCNA (#13110), anti-YAP (#14074), and anti-CREB (#9197), all diluted 1:200 and purchased from CST, and rat anti-Actin (#ab130935) was diluted 1:200 and purchased from Abcam.

### CUT & RUN assay

Protein-DNA interactions were analysed using cleavage under targets and release using a nuclease (CUT&RUN) assay kit (#86652; CST) following the manufacturer’s instructions. Approximately 100,000 cells were used for each reaction at room temperature. The positive control was a rabbit monoclonal antibody (mAb) against trimethylated histone H3 (Lys4) (H3K4me3; #9751), and the negative control was a rabbit mAb IgG isotype (#66362), which was provided in the kit. They were used with an anti-CREB antibody (diluted 1:50; #9197; CST) for binding and the subsequent cleavage and release steps. The extracted DNA was purified using DNA purification buffers and spin columns (#14209; CST). Finally, the DNA products were quantified by qPCR and DNA agarose gel electrophoresis, and the primers used are shown in Additional file 1: Table S3.

### Dual-luciferase reporter assay

The CREB control plasmid, CREB plasmid, firefly luciferase plasmid with the YAP-CREB-binding site (CBS) wild-type promoter, firefly luciferase plasmid with the YAP-CBS mutation promoter and Renilla luciferase plasmid were all purchased from Syngentech (Beijing Syngentech Co., Ltd., Beijing, China) and used to transfect HEK293T cells (# CTCC-001-0188; Meisen Chinese Tissue Culture Collections, Zhejiang, China). Next, the luciferase activity was analysed using a dual-luciferase reporter kit (# RG027; Beyotime) and an iMark Microplate Absorbance Reader (Bio–Rad Laboratories, Inc., CA, USA). The firefly luciferase data were normalized to the Renilla luciferase data and expressed as the fold change relative to the control. The dual-luciferase reporter assays were conducted for six replicates per group and repeated independently three times.

### Statistical analysis

The data for all experiments were shown as means ± standard deviation (SD) of three biological replicates, and all data analyses were performed using GraphPad Prism 7 software (San Diego, CA). Statistical analysis between groups was performed using Student’s *t* test to determine significance. *P value*s < 0.05 were considered statistically significant.

## Results

### RAMP1 overexpression promotes MSF proliferation

**Fig. 1:**
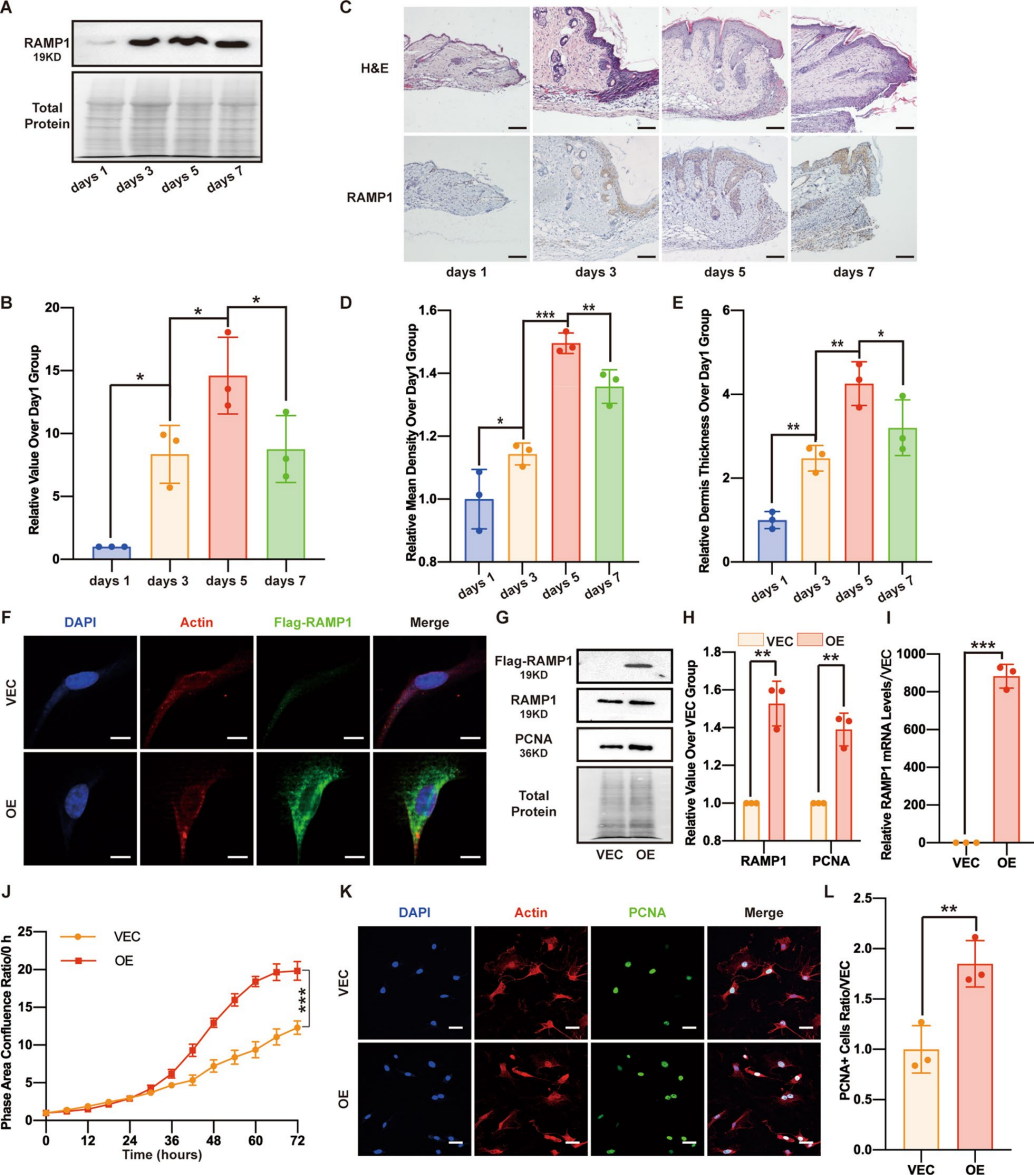
RAMP1 overexpression promotes cell proliferation. **A** Western blot analysis of RAMP1 in skin wound tissue proteins. Total protein was stained as an endogenous control. **B** Quantification of the protein band intensities in **A**. The data were normalized by lane normalization factor (LNF). **C** H&E staining and IHC staining of RAMP1 in skin wound tissue sections (10×). Scale bar = 100 μm. **D** Quantitative analysis of RAMP1 expression by IHC staining of **C**. **E** Dermis thickness measurements by H&E staining of **C**. RAMP1 OE and VECs were treated with DOX (5 μg/ml) for 48 h when the following experiments were conducted. **F** Confocal immunofluorescence of Flag (green), Actin (red) and DAPI (blue). Scale bar = 10 μm. **G** Western blot analysis of Flag-RAMP1, RAMP1 and PCNA. Total protein was stained as an endogenous control. **H** Quantification of the protein band intensities of **G**. The data were normalized by LNF. **I** qPCR analysis of RAMP1 mRNA levels. The data were normalized to the amount of β-actin mRNA. **J** Cell proliferation ability (72 h) measured by confluence measurements normalized to hour 0 calculated using IncuCyte (Essen BioScience). **K** Confocal immunofluorescence of PCNA (green), Actin (red) and DAPI (blue). Scale bar = 20 μm. **L** Dot plots show quantification of the frequency of PCNA-positive cells in each field of view. The experiments were performed in triplicate. The data are presented as means ± SD, and significant differences were evaluated using unpaired *t test*. *P < 0.05, **P < 0.01 and ***P < 0.005

To investigate the changes in RAMP1 expression during skin wound healing, we first performed mouse skin wound tissue protein analysis using Western blotting and IHC staining (Fig. 1A–D). The RAMP1 content and mean optical density of RAMP1 in the dermis gradually increased from 24 h (days 1) to days 3 and days 5 post-injury. By days 7, when the wound area was healed by approximately 95%, the RAMP1 content decreased compared with that on days 5 but was still higher than that at 24 h (days 1). The dermis thickness measurements by H&E staining of the skin sections also showed the same trend (Fig. 1C, E). Given that fibroblasts are one of the most important cells of the dermis and in the wound healing process, we next explored the role of RAMP1 in MSF function. First, we infected MSFs with Tet-On-Flag-RAMP1 lentivirus and Tet-On-Flag-vector lentivirus. After DOX (5 μg/ml) induction for 48 h, IF showed Flag expression in both the vector control (VEC) and RAMP1 overexpression (OE) group, and the fluorescence of the OE group was more intense (Fig. 1F). Additionally, Western blot and qPCR analyses were performed to assess infection success, revealing that RAMP1 protein expression and mRNA transcription in the OE group were significantly higher than those in the VEC group (Fig. 1G–I). After successfully infecting RAMP1-overexpressing MSFs and vector MSFs, we conducted an MSF proliferation assay using an IncuCyte S3 system. The rate at which the MSFs reached confluence in an area was much faster in the OE group than in the VEC group (Fig. 1J). To confirm the reliability of the S3 results based on cell confluence area measurement, we examined PCNA expression in both groups by performing Western blotting and IF staining. As expected, PCNA expression was higher in the OE group than in the VEC group (Fig. 1G, H, K, L), consistent with the S3 proliferation results, indicating that RAMP1 upregulation can promote the proliferation ability of MSFs.

### RAMP1 overexpression increases Gαi3, PKA, CREB and YAP expression

**Fig. 2:**
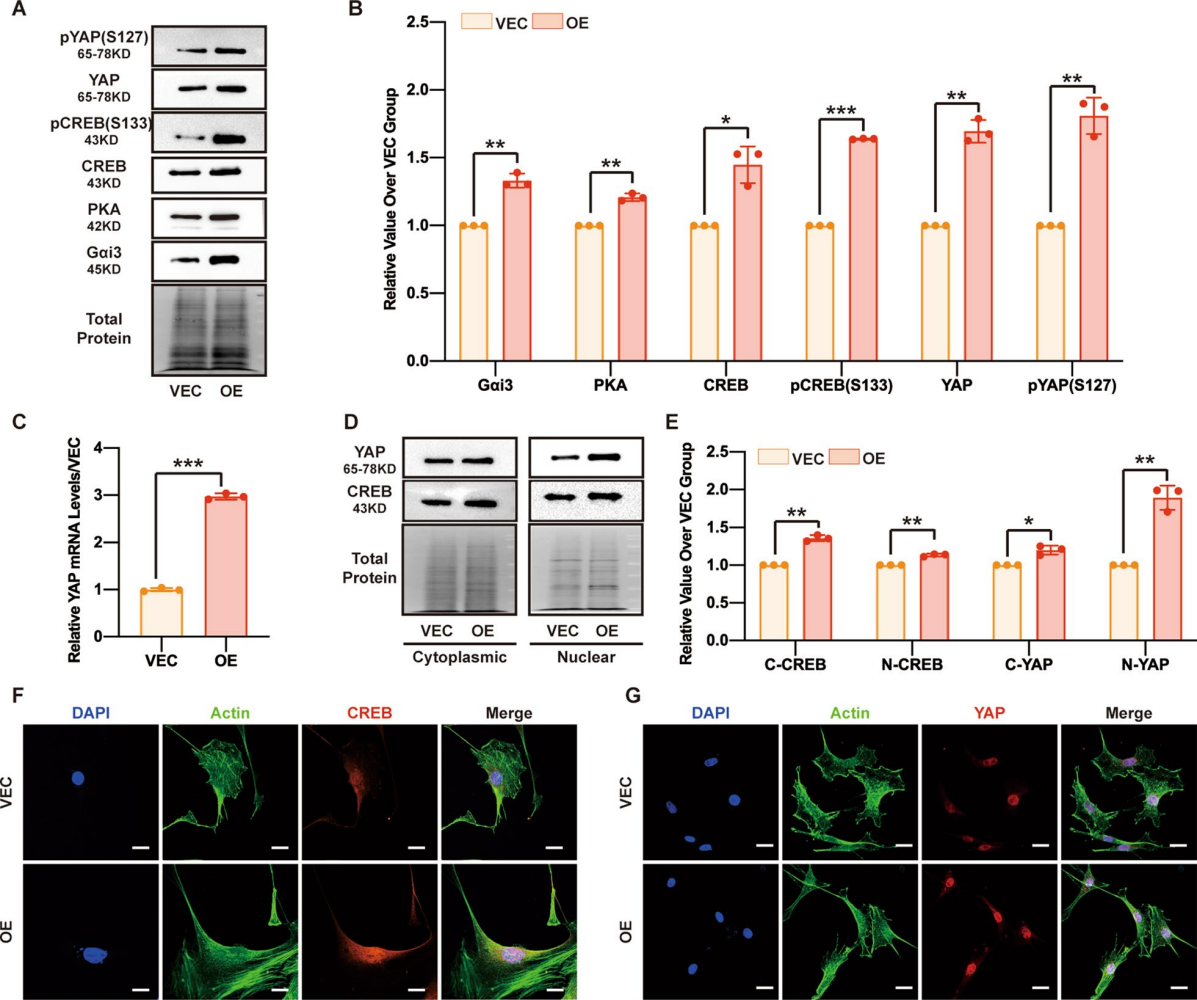
RAMP1 overexpression increases Gαi3, PKA, CREB and YAP expression. RAMP1 OE and VECs were treated with DOX (5 μg/ml) for 48 h when the following experiments were conducted. **A** Western blot analysis of Gαi3, PKA, CREB, pCREB (S133), YAP and pYAP (S127). Total protein was stained as an endogenous control. **B** Quantification of the protein band intensities in **A**. The data were normalized to LNF. **C** qPCR analysis of the YAP mRNA levels. The data were normalized to the amount of β-actin mRNA. **D** Western blot analysis of cytoplasmic and nuclear fractions of CREB and YAP. Total protein was stained as an endogenous control. **E** Quantification of the protein band intensities in **D**. The data were normalized to LNF. C-CREB means cytoplasmic CREB, N-CREB means nuclear CREB, C-YAP means cytoplasmic YAP, and N-YAP means nuclear YAP. **F** Confocal immunofluorescence of CREB (red), Actin (green) and DAPI (blue). Scale bar = 20 μm. **G** Confocal immunofluorescence of YAP (red), Actin (green) and DAPI (blue). Scale bar = 20 μm. The experiments were performed in triplicate. The data are presented as means ± SD, and significant differences were evaluated using unpaired *t test*. *P < 0.05, **P < 0.01 and ***P < 0.005

Because RAMP1 is an upstream factor in the context of the entire proliferation pathway, we explored the pathway downstream of regulatory RAMP1 during MSF proliferation to determine whether YAP is part of this pathway. We first evaluated YAP and pYAP (S127) in the MSFs of the OE and VEC groups. The expression of both YAP and pYAP (S127) was elevated when RAMP1 was overexpressed (Fig. 2A, B). qPCR analysis indicated that the YAP transcription level was also elevated in the OE group (Fig. 2C). YAP expression was higher in both the cytoplasmic and nuclear extracts in the OE group than in the VEC group (Fig. 2D, E, G). Considering that RAMP1 can interact with and modulate the activities of GPCRs and that YAP can be regulated by signalling mediated by GPCRs, we next examined the G protein subunits and classical factors in the pathway downstream of GPCRs. The expression of Gαi3, PKA, CREB and its active form, pCREB (S133), was upregulated in the OE group (Fig. 2A, B). Additionally, subcellular fractionation was assessed by Western blotting and IF staining, and CREB expression was elevated in both the cytoplasm and nucleus (Fig. 2D–F). These data demonstrated that RAMP1 OE led to an increase in Gαi3, PKA, CREB, pCREB (S133), YAP and pYAP (S127) expression and that this effect may involve an axis downstream of regulatory RAMP1 during MSF proliferation.

### RAMP1 overexpression promotes proliferation by increasing YAP protein levels

**Fig. 3:**
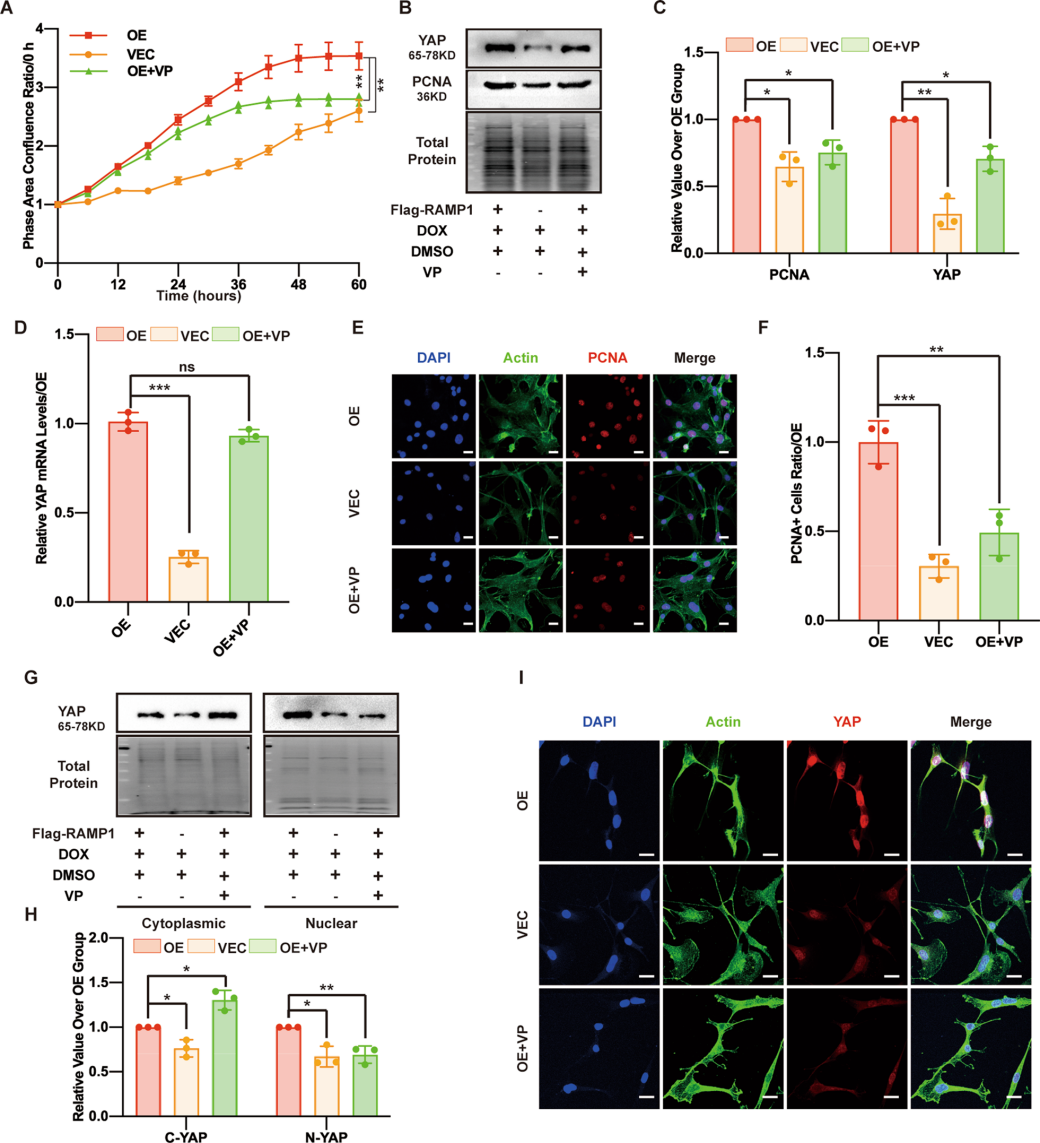
RAMP1 overexpression promotes proliferation by increasing YAP protein levels. In the OE+VP group, RAMP1 OE cells were first treated with veterporfin (VP, 0.01 μM) alone for 12 h and then with VP (0.01 μM) and DOX (5 μg/ml) for 48 h. In the OE and VEC groups, RAMP1 OE and VECs were first treated with DMSO (0.01 μM) alone for 12 h and then with DMSO (0.01 μM) and DOX (5 μg/ml) for 48 h. **A** Cell proliferation ability measured by confluence measurements normalized to hour 0  and calculated using IncuCyte (Essen BioScience). **B** Western blot analysis of PCNA and YAP. Total protein was stained as an endogenous control. **C** Quantification of the protein band intensities in **B**. The data were normalized to LNF. **D** qPCR analysis of the YAP mRNA levels. The data were normalized to the amount of β-actin mRNA. **E** Confocal immunofluorescence of PCNA (red), Actin (green) and DAPI (blue). Scale bar = 20 μm. **F** The dot plots show quantification of the frequency of PCNA-positive cells in each field of view. **G** Western blot analysis of the cytoplasmic and nuclear fractions of YAP. Total protein was stained as an endogenous control. **H** Quantification of the protein band intensities of **G**. The data were normalized to LNF. C-YAP means cytoplasmic YAP, and N-YAP means nuclear YAP. **I** Confocal immunofluorescence of YAP (red), Actin (green) and DAPI (blue). Scale bar = 20 μm. All the experiments were performed in triplicate. The data are presented as means ± SD, and significant differences were evaluated using unpaired *t test*. *P < 0.05, **P < 0.01 and ***P < 0.005

To elucidate whether the elevated expression and activity of YAP promoted MSF proliferation, verteporfin (VP, 0.01 μM), a YAP inhibitor, was added during the culture of the MSF OE group (OE + VP group). After VP treatment, the MSF proliferation rate of the OE+VP group was significantly decreased compared with that of the RAMP1 OE group (Fig. 3A). Western blotting and IF detection of PCNA also confirmed that the PCNA level was reduced in the OE + VP group (Fig. 3B, C, E, F). VP treatment also decreased the total YAP protein level in the OE + VP group (Fig. 3B, C). Cytoplasmic and nuclear extraction Western blotting and IF showed that cytoplasmic YAP and nuclear YAP levels were also decreased in the OE + VP group (Fig. 3G–I); however, qPCR analysis showed that VP treatment could not influence YAP transcription (Fig. 3D). Thus, VP directly prevented the YAP-TEAD interaction and its downstream signalling, leading to YAP sequestration in the cytoplasm and degradation, but did not directly prevent YAP protein expression [37]. In addition to the in vitro findings, the in vivo application of VP (100 nmol) to mouse wounds delayed wound healing (Additional file 2: Fig. S1A and S1B), indicating that YAP inhibition could ameliorate wound healing. The data confirmed that YAP is the downstream effector of RAMP1 regulation of MSF proliferation.

### Interference with Gαi3 after RAMP1 overexpression decreases PKA, CREB and YAP expression and MSF proliferation

**Fig. 4:**
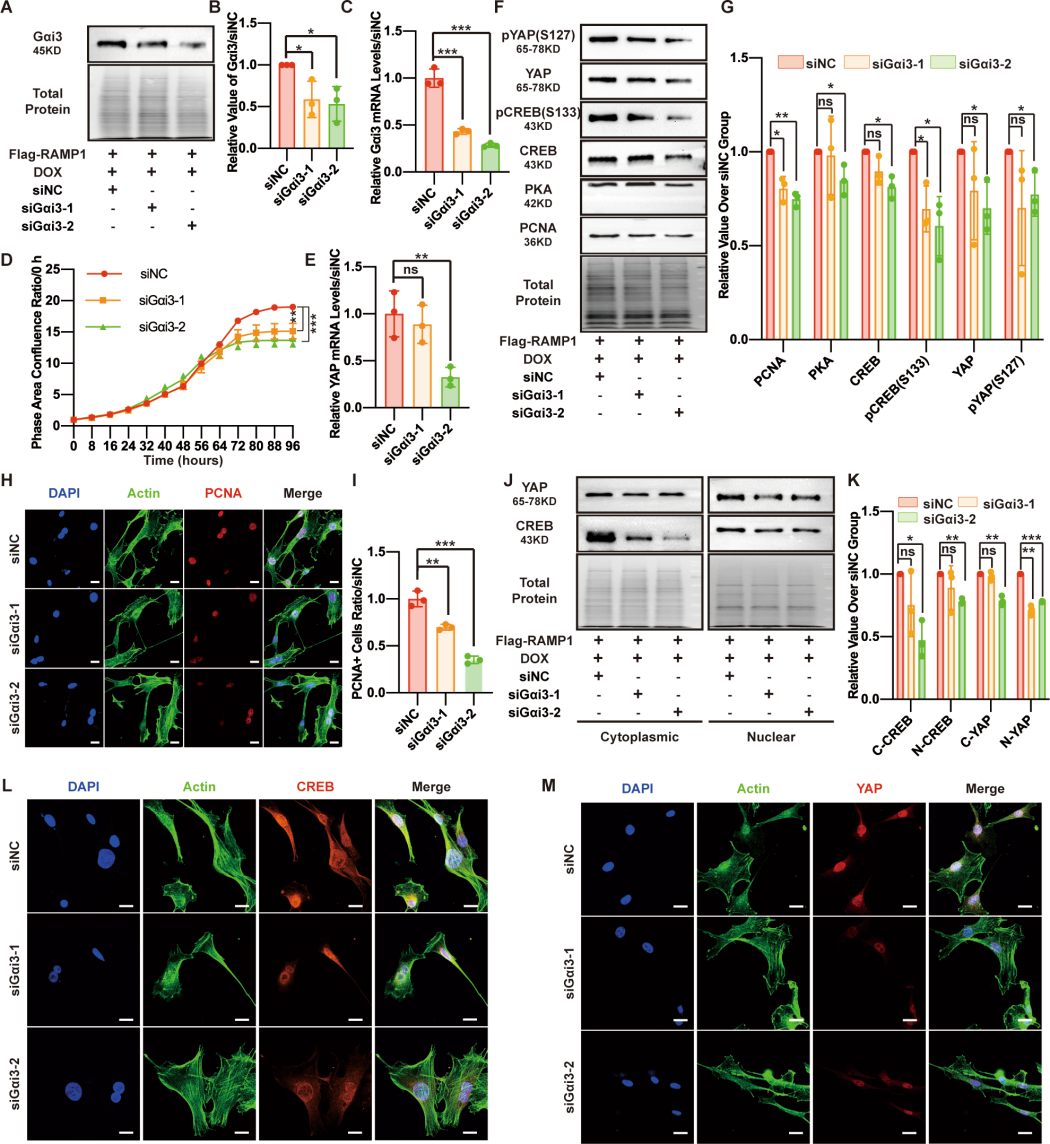
Interference with Gαi3 after RAMP1 overexpression decreases PKA, CREB, and YAP expression and MSF proliferation. RAMP1 OE cells were reverse-transfected with 50 nM of small interfering RNAs (siRNAs) specifically targeting Gαi3 (siGαi3-1 and siGαi3-2) or a negative control sequence (siNC) for 48 h and then were treated with DOX (5 μg/ml) for 48 h. **A** Western blot analysis of Gαi3. Total protein was stained as an endogenous control. **B** Quantification of the protein band intensities of **A**. The data were normalized to LNF. **C** qPCR analysis of the Gαi3 mRNA levels. The data were normalized to the amount of β-actin mRNA. **D** Cell proliferation ability measured by confluence measurements normalized to hour 0 calculated using IncuCyte (Essen BioScience). **E** qPCR analysis of the YAP mRNA levels. The data were normalized to the amount of β-actin mRNA. **F** Western blot analysis of PCNA, PKA, CREB, pCREB (S133), YAP, and pYAP (S127). Total protein was stained as an endogenous control. **G** Quantification of the protein band intensities in **F**. The data were normalized to LNF. **H** Confocal immunofluorescence of PCNA (red), Actin (green) and DAPI (blue). Scale bar = 20 μm. **I** Dot plots show quantification of the frequency of PCNA-positive cells in each field of view. **J** Western blot analysis of the cytoplasmic and nuclear fractions of CREB and YAP. Total protein was stained as an endogenous control. **K** Quantification of the protein band intensities of **J**. The data were normalized to LNF. C-CREB means cytoplasmic CREB, N-CREB means nuclear CREB, C-YAP means cytoplasmic YAP, and N-YAP means nuclear YAP. **L** Confocal immunofluorescence of CREB (red), Actin (green) and DAPI (blue). Scale bar = 20 μm. **M** Confocal immunofluorescence of YAP (red), Actin (green) and DAPI (blue). Scale bar = 20 μm. The experiments were performed in triplicate. The data are presented as means ± SD, and significant differences were evaluated using unpaired *t test*. *P < 0.05, **P < 0.01, ***P < 0.005 and ns, not significant

To identify the mechanism by which RAMP1 regulates YAP expression, Gαi3 siRNA was first used to silence Gαi3 activity of RAMP1 OE cells in the siGαi3-1 and siGαi3-2 groups, while a negative control sequence was used in the siNC group as a control (Fig. 4A–C). Gαi3 interference decreased the proliferation rate of MSFs (Fig. 4D), and the Western blot and IF staining analyses of PCNA expression also demonstrated the inhibitory effect of Gαi3 interference on MSF proliferation (Fig. 4F–I). Next, we detected the downstream protein levels, revealing that PKA, CREB and YAP levels were downregulated after Gαi3 interference and the YAP transcription level was also decreased (Fig. 4E–G). The level of pCREB (S133), which is the active form of CREB, was decreased in the siGαi3-1 group and siGαi3-2 group. Additionally, the level of pYAP (S127), which is the inactive form of YAP, was decreased in both Gαi3 interference groups (Fig. 4F, G). Because both YAP and CREB function in the nucleus, we performed Western blotting and IF staining on the extracted cytoplasmic and nuclear fractions to identify the nuclear protein level. Both YAP and CREB levels in the nucleus were significantly lower in the siGαi3-1 and siGαi3-2 groups than in the siNC group, and Gαi3 silencing led to reduced cytoplasmic localization of YAP and CREB (Fig. 4J–M). The nonhealing rate of wounds on the siGαi3-2 (5 nmol) side at 24 h (days 1), days 3, days 5 and days 7 was significantly higher than that of wounds on the siNC side at the corresponding time points (Additional file 2: Fig. S1C and S1D), indicating that inhibition of Gαi3 delayed the healing of mouse wounds. These results suggest that RAMP1 may regulate YAP by modulating Gαi3 expression.

### The PKA inhibitor H-89 reduces CREB and YAP levels and inhibits MSF proliferation

**Fig. 5:**
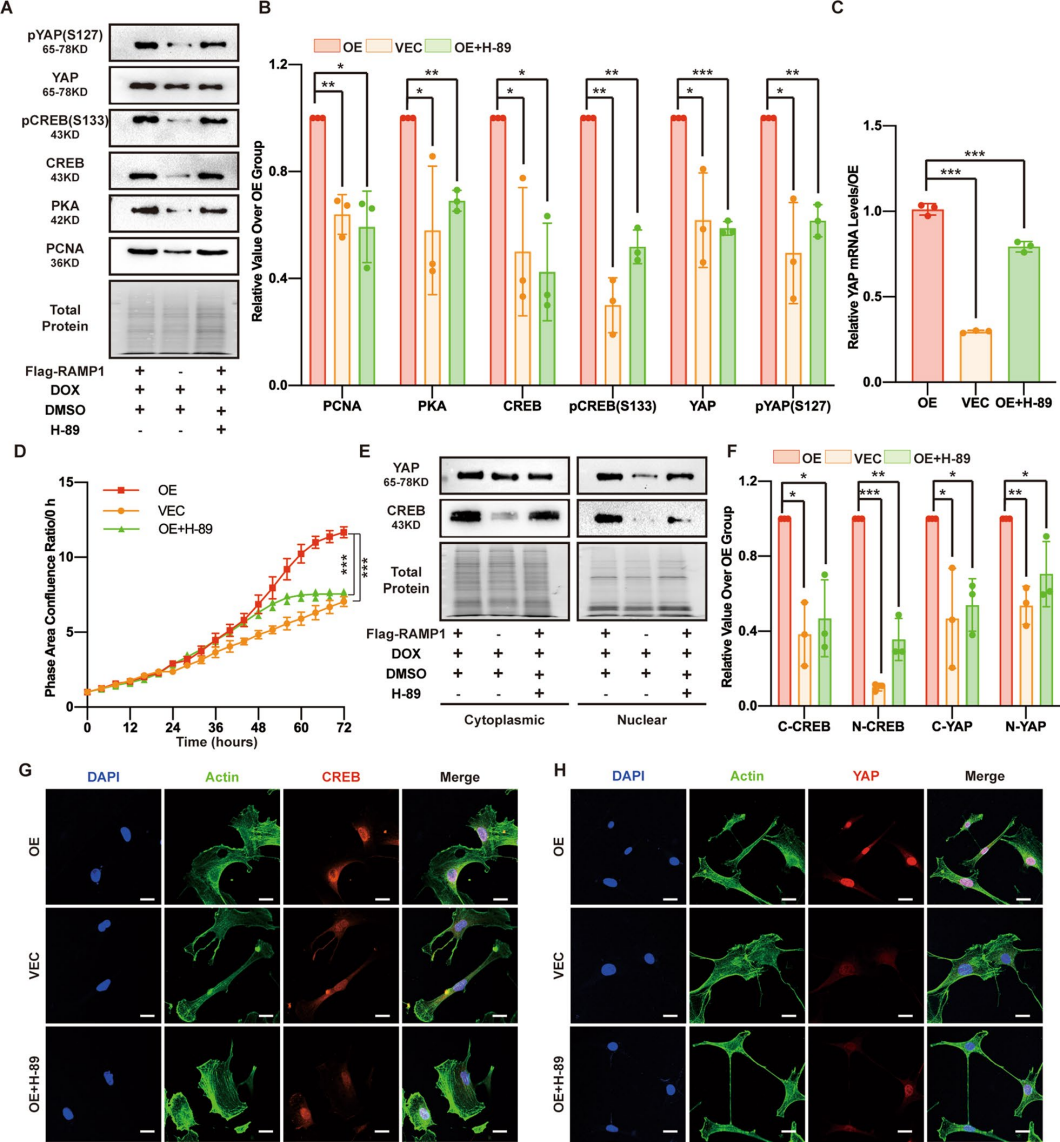
The PKA inhibitor H-89 reduces CREB and YAP levels and inhibits MSF proliferation. In the OE+H-89 group, RAMP1 OE cells were first treated with H-89 (1 μM) alone for 24 h and then with H-89 (1 μM) and DOX (5 μg/ml) for 48 h. In the OE and VEC groups, RAMP1 OE and VECs were first treated with DMSO (1 μM) alone for 24 h and then with DMSO (1 μM) and DOX (5 μg/ml) for 48 h. **A** Western blot analysis of PCNA, PKA, CREB, pCREB (S133), YAP and pYAP (S127). Total protein was stained as an endogenous control. **B** Quantification of the protein band intensities in **A**. The data were normalized to LNF. **C** qPCR analysis of the YAP mRNA levels. The data were normalized to the amount of β-actin mRNA. **D** Cell proliferation ability measured by confluence measurements normalized to hour 0 and calculated using IncuCyte (Essen BioScience). **E** Western blot analysis of the cytoplasmic and nuclear fractions of YAP and CREB. Total protein was stained as an endogenous control. **F** Quantification of the protein band intensities of **E**. The data were normalized to LNF. C-CREB means cytoplasmic CREB, N-CREB means nuclear CREB, C-YAP means cytoplasmic YAP, and N-YAP means nuclear YAP. **G** Confocal immunofluorescence of CREB (red), Actin (green) and DAPI (blue). Scale bar = 20 μm. **H** Confocal immunofluorescence of YAP (red), Actin (green) and DAPI (blue). Scale bar = 20 μm. The experiments were performed in triplicate. The data are presented as means ± SD, and significant differences were evaluated using unpaired *t test*. *P < 0.05, **P < 0.01 and ***P < 0.005

Many signalling molecules might participate in the processes between RAMP1-Gαi3 axis activation and YAP activity regulation. Because PKA, CREB and pCREB (S133) were upregulated in the RAMP1 OE group and downregulated in the Gαi3 interference group, we asked whether PKA is a downstream factor of Gαi3 and an upstream factor of YAP. By treating the RAMP1 OE group with the PKA inhibitor H-89 dihydrochloride (H-89; 1 μM; OE+H-89 group), we observed a diminished proliferation rate in the S3 cell proliferation analysis and consistently decreased PCNA expression by Western blotting (Fig. 5A, B, D). H-89 also reduced the PKA, CREB and YAP protein levels and YAP transcription levels, and pCREB (S133) and pYAP (S127) were downregulated in the OE + H-89 group (Fig. 5A–C). Additionally, we measured the YAP and CREB protein levels in both the cytoplasmic and nuclear extract fractions and by IF staining to determine the effect of H-89 on effector localization. H-89 reduced the YAP and CREB levels in the nucleus and cytoplasm (Fig. 5E–H). The optical images and statistical analysis showed that topical continuous treatment with H-89 decreased the wound healing rate compared with that of the DMSO control group, particularly in the early phase (Additional file 2: Fig. S1E and S1F), indicating that H-89 treatment may delay wound healing by inhibiting PKA. These results indicate that RAMP1-Gαi3 may regulate PKA function to modulate downstream effects.

### The PKA agonist BUC increases CREB and YAP expression and MSF proliferation

**Fig. 6:**
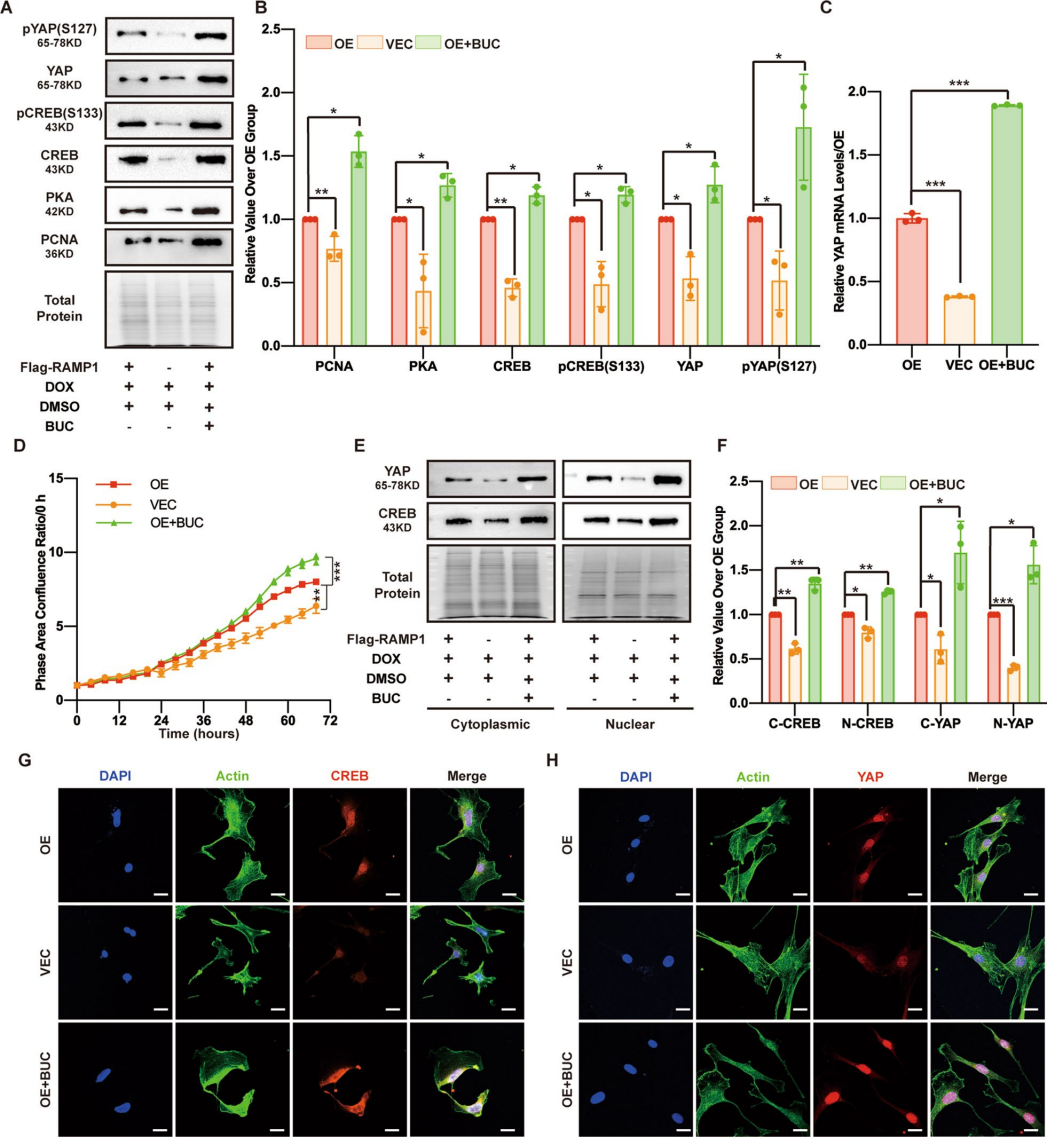
The PKA agonist BUC increases CREB and YAP expression and MSF proliferation. In the OE+BUC group, RAMP1 OE cells were first treated with DOX (5 μg/ml) for 48 h and then with BUC (0.01 μM) and DOX (5 μg/ml) for 24 h. In the OE and VEC groups, RAMP1 OE and VECs were first treated with DOX (5 μg/ml) for 48 h and then DMSO (0.01 μM) and DOX (5 μg/ml) for 24 h. **A** Western blot analysis of PCNA, PKA, CREB, pCREB (S133), YAP and pYAP (S127). Total protein was stained as an endogenous control. **B** Quantification of the protein band intensities in **A**. The data were normalized to LNF. **C** qPCR analysis of the YAP mRNA levels. The data were normalized to the amount of β-actin mRNA. **D** Cell proliferation ability measured by confluence measurements normalized to hour 0 and calculated using IncuCyte (Essen BioScience). **E** Western blot analysis of the cytoplasmic and nuclear fractions of YAP and CREB. Total protein was stained as an endogenous control. **F** Quantification of the protein band intensities of **E**. The data were normalized to LNF. C-CREB means cytoplasmic CREB, N-CREB means nuclear CREB, C-YAP means cytoplasmic YAP, and N-YAP means nuclear YAP. **G** Confocal immunofluorescence of CREB (red), Actin (green) and DAPI (blue). Scale bar = 20 μm. **H** Confocal immunofluorescence of YAP (red), Actin (green) and DAPI (blue). Scale bar = 20 μm. The experiments were performed in triplicate. The data are presented as means ± SD, and significant differences were evaluated using unpaired *t test*. *P < 0.05, **P < 0.01 and ***P < 0.005

We used the PKA agonist BUC (0.01 μM) to identify the role of PKA in YAP activity regulation and proliferation rate of RAMP1-overexpressing cells. Consistently, BUC elevated the MSF proliferation rate, as evaluated by the cell confluence area ratio measured by the S3 instrument and by the expression of the proliferation marker PCNA as measured by Western blotting (Fig. 6A, B, D). To determine downstream PKA, CREB and YAP protein levels and YAP transcription levels, BUC treatment was administered, revealing a positive effect (Fig. 6A–C). Interestingly, BUC increased the level of the CREB active form, pCREB (S133), and the YAP inactive form, pYAP (S127) (Fig. 6A, B). Next, we explored the effect of BUC on CREB and YAP cell localization, revealing that BUC caused increased cytoplasmic and nuclear localization of YAP and CREB (Fig. 6E–H). We also observed that the wound closure percentage of the BUC (100 nmol)-treated side was higher at each corresponding observation time point than that of the DMSO-treated side (Additional file 2: Fig. S1G and S1H), indicating that stimulation of PKA by BUC may promote the wound healing rate. Taken together, these results indicate that RAMP1-Gαi3 modulates MSF proliferation by regulating PKA function, but the pathway components remain to be identified.

### The RAMP1-Gαi3-PKA axis regulates YAP mediated by CREB

**Fig. 7:**
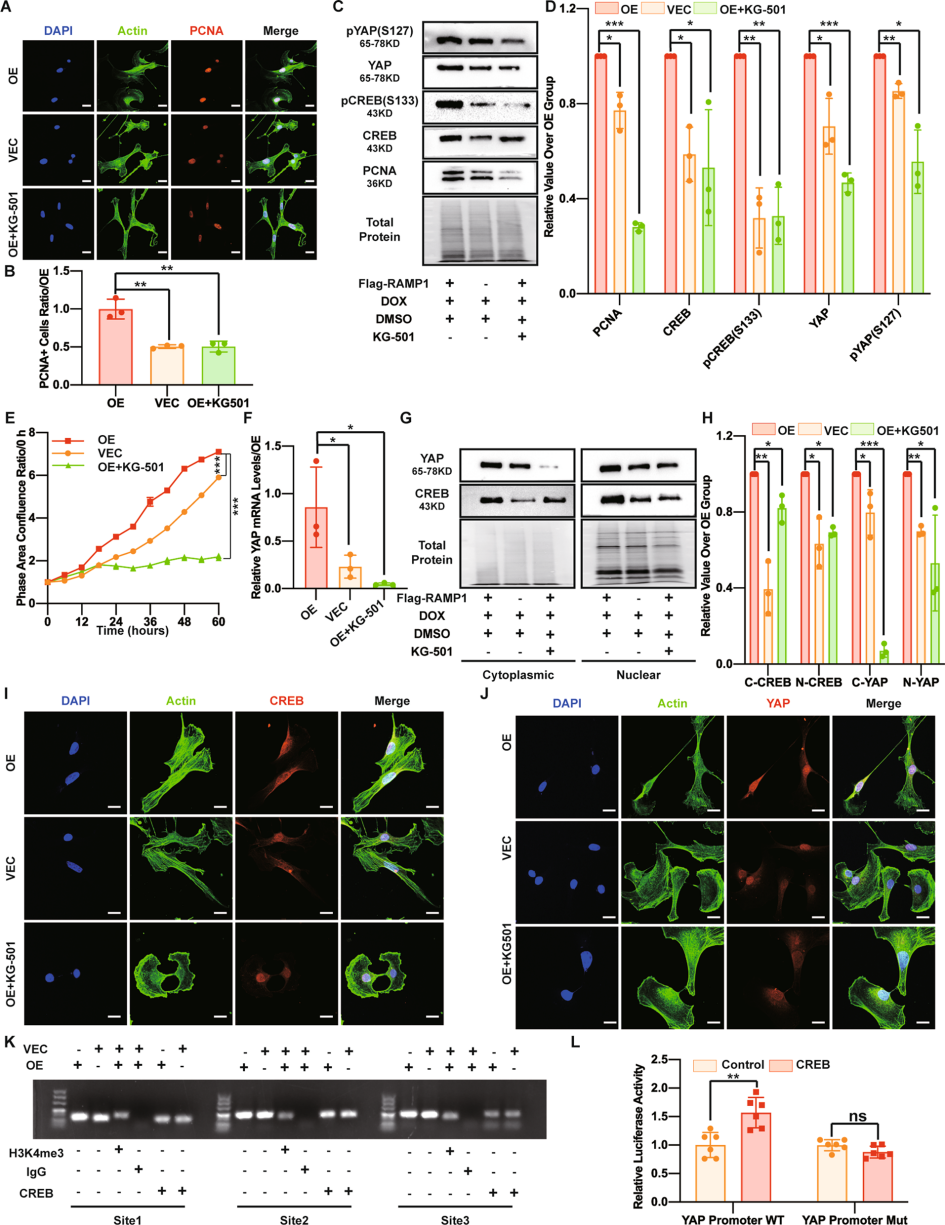
The RAMP1-Gαi3-PKA axis regulates YAP mediated by CREB. In the OE+KG-501 group, RAMP1 OE cells were first treated with KG-501 (10 μM) for 12 h and then with KG-501 (10 μM) and DOX (5 μg/ml) for 48 h. In the OE and VEC groups, RAMP1 OE and VECs were first treated with DMSO (10 μM) for 12 h and then with  DMSO (10 μM) and DOX (5 μg/ml) for 48 h. **A** Confocal immunofluorescence of PCNA (red), Actin (green) and DAPI (blue). Scale bar = 20 μm. **B** Dot plots show quantification of the frequency of PCNA-positive cells in each field of view. **C** Western blot analysis of PCNA, CREB, pCREB (S133), YAP and pYAP (S127). Total protein was stained as an endogenous control. **D** Quantification of the protein band intensities in **C**. The data were normalized to LNF. **E** Cell proliferation ability measured by confluence measurements normalized to hour 0 and calculated using IncuCyte (Essen BioScience). **F** qPCR analysis of the YAP mRNA levels. The data were normalized to the amount of β-actin mRNA. **G** Western blot analysis of the cytoplasmic and nuclear fractions of YAP and CREB. Total protein was stained as an endogenous control. **H** Quantification of the protein band intensities of **G**. The data were normalized to LNF. C-CREB means cytoplasmic CREB, N-CREB means nuclear CREB, C-YAP means cytoplasmic YAP, and N-YAP means nuclear YAP. **I** Confocal immunofluorescence of CREB (red), Actin (green) and DAPI (blue). Scale bar = 20 μm. **J** Confocal immunofluorescence of YAP (red), Actin (green) and DAPI (blue). Scale bar = 20 μm. **K** DNA agarose gel electrophoresis of *YAP* promoter DNA quantification using the CUT&RUN assay and qPCR with different primers. **L** Dual-luciferase reporter assay of the CBS wild-type (WT) *YAP* promoter and mutated (Mut) YAP promoter. The experiments were performed in triplicate. The data were presented as means ± SD, and significant differences were evaluated using unpaired *t test*. *P < 0.05, **P < 0.01, ***P < 0.005 and ns, not significant

Because CREB is the most common downstream factor of PKA and the abovementioned results showed that CREB is regulated by the RAMP1-Gαi3-PKA axis, we asked whether CREB can regulate YAP levels and whether this regulation is accomplished at the transcriptional level. To identify the regulatory effect of CREB on YAP, we first administered KG-501 (10 μM), a CREB activity inhibitor, to RAMP1-OE cells and observed a decreased proliferation rate by the S3 assay and Western blotting and IF staining for PCNA (Fig. 7A–E). As speculated, KG-501 diminished YAP and pYAP (S127) levels in the OE+KG-501 group. Interestingly, although KG-501 was first found to function by inhibiting the binding of CREB [38], we also observed that the CREB and pCREB (S133) levels were reduced by KG-501 (Fig. 7C, D) and the level of cytoplasmic CREB and that of nuclear CREB was reduced (Fig. 7G–I). Additionally, KG-501 treatment led to a decrease in both cytoplasmic and nuclear YAP levels and reduced YAP mRNA transcription (Fig. 7F, G, H, J). Next, we evaluated CREB transcriptional activity in relation to YAP levels. The JASPAR database was used to analyse CBSs in *YAP* promoter sequences (Additional file 3: Fig. S2A). The three candidate sites with the highest predicted score were − 818 to 825 bp, − 2664 to 2671 bp and − 1204 to 1211 bp in *YAP* promoter (Additional file 1: Table S4). To determine whether CREB binds at these sites to target *YAP *promoter, we performed a CUT&RUN assay, and the qPCR and agarose gel electrophoresis results confirmed that CREB can directly bind to the *YAP* promoter at these three sites (Additional file 3: Fig. S2B; Fig. 7K). Next, to determine whether these candidate sites are cAMP responsive elements (CREs), we performed a luciferase promoter activity assay, revealing that CREB could transactivate the wild-type reporter construct but failed to transactivate the mutation reporter with the deletion of the above three predicted binding sites, indicating that CREB can act on at least one of the three sites on the *YAP* promoter region (Fig. 7L). Further experiments are needed to identify which sites are the most functional. We also performed an in vivo experiment by administering KG-501 (100 nmol) on skin wounds on the right side and found that KG-501 significantly slowed the wound closure rate (Additional file 2: Fig. S1I and S1J) compared with the control group on the other side, indicating that the CREB inhibitor KG-501 could slow wound healing, particularly at the early stage. Together, these results indicate that the RAMP1-Gαi3-PKA axis can regulate YAP levels and MSF proliferation mediated by CREB and that CREB can bind to the *YAP* promoter and promote YAP transcription.

## Discussion

Abnormal skin innervation can lead to abnormal skin wound healing, which includes an excessive wound healing process, such as scarring, or inadequate wound healing, such as the formation of chronic nonhealing ulcers and ischaemic chronic wounds [6, 9, 14, 39, 40]. CGRP-positive nerve endings are among the most widely distributed peptidergic nerve fibres and play a crucial role in normal wound healing [8, 40]. However, CGRP causes many side effects directly in wounds [41, 42]. Recently, RAMP1 was reported to promote wound healing by increasing angiogenesis and lymphangiogenesis [20], suggesting that CGRP function can be modulated by manipulating RAMP1. However, few studies on RAMP1 and skin cells have been performed; therefore, it is necessary to explore the function of RAMP1 in skin cells and verify the downstream regulatory mechanism.

In the present study, we first identified that RAMP1 levels change during wound healing, suggesting that RAMP1 functions during the wound healing process. Next, we successfully constructed a RAMP1-overexpressing MSF cell line in vitro and found that it can promote MSF proliferation. By performing mechanistic studies, we found that the effect of RAMP1 OE on MSF proliferation was mainly mediated by the Gαi3-PKA-CREB-YAP axis, leading to upregulated transcription of YAP, increased nuclear YAP levels and an elevated MSF proliferation rate. In vivo experiments confirmed the role of Gαi3-PKA-CREB-YAP axis molecules in wound healing.

RAMP1 is a member of the RAMP family, which includes two other identified members, RAMP2 and RAMP3 [19, 43]. RAMP1 was first identified during an effort to understand CGRP signalling, and it can modify CLR downstream signalling, traffic CLR to the cell surface, and affect CLR internalization [43]. However, the distribution of RAMP1 is wider than that of CLR [43, 44], and Debbie et al. reported that the cortex and hippocampus expressed RAMP1 but not CLR [45], indicating that RAMP1 has functions other than those related to CLR. Previous studies of RAMP1 focused primarily on its effect on exogenous or endogenous CGRP function, and the function of RAMP1 alone on fibroblasts has not been previously explored. In this study, we explored, without using exogenous CGRP, whether RAMP1 alone can affect MSF function. We observed that RAMP1 OE alone can promote MSF proliferation, indicating, for the first time, that RAMP1 alone can exert an effect, but exploration into its downstream regulatory mechanism is necessary.

RAMPs have been reported to promote G protein uncoupling [46] and alter the association of GPCR coupling to G proteins, in addition to Gαs, Gαq, and Gαi [47]. We found that with RAMP1 OE, the Gαi3 level was increased, and the downstream PKA and CREB levels were elevated. Gαi3 is one of the three inhibitory subunits of G proteins that can inhibit AC activity, leading to decreased cAMP production and PKA activity [48]. However, Gαi3 has also been shown to stimulate AC2 and AC4 to increase cAMP levels and PKA activity [29]. The current study indicated that RAMP1 OE can upregulate Gαi3 to promote PKA activity, but whether it was performed by stimulating AC2 or AC4 remains to be verified in our subsequent study.

PKA has been reported to phosphorylate LATS1/2 and thus phosphorylate YAP, which mostly occurs at S127 and leads to Hippo pathway activation [30, 31, 49], and we observed elevated pYAP (S127), consistent with a previously reported study. Although the elevated Ser127 phosphorylation of YAP has been linked to the functional inactivation of YAP through cytoplasmic retention and degradation [50, 51] and we found that the cytoplasmic YAP level was higher in the RAMP1 OE group than in the control group, we observed that the nuclear YAP level was also higher in the OE group, indicating that the total YAP protein level was elevated. In other words, the transcription and synthesis of YAP were both increased; hence, although YAP was retained in the cytoplasm, YAP translocation into the nucleus remained high. CREB has been reported to be a transcription factor of YAP in hepatoma cells [32, 52]. In our study, using the CUT&RUN assay and dual-luciferase reporter assays, CREB could bind to sites in the YAP promoter and functioned as a transcription factor. We clarify that CREB is a YAP transcription factor in MSFs, but the specific site must be verified using dual luciferase reporter assays at different mutation sites.

CREB, a 43 kDa amino acid, is the classic downstream factor of PKA. As a transcription factor, it functions by binding to the CRE with coactivators, such as CREB-binding protein (CBP), to activate the downstream transcriptional signal [53, 54]. Phosphorylation of amino acid residues is the regulatory mechanism of CREB, and different phosphorylation patterns serve different functions, among which the Ser133 residue frequently occurs and induces transcription, while Ser111 occurs less frequently and inhibits transcription [54, 55]. In our study, elevated CREB and pCREB (S133) were observed in total protein extracts and subcellular protein extracts, indicating that activated CREB was a downstream factor of RAMP1 OE. KG-501 was first identified by Best et al. [38] as a CREB-CBP inhibitor, and Steven A et al. [56] reported that KG-501 could decrease Her-2/neu-overexpressing cell migration without influencing CREB expression and phosphorylation. In our study, KG-501 treatment did not influence the CREB transcription level (data not shown); however, we also observed decreased CREB protein levels not only in total protein extracts but also in cytoplasmic and nuclear proteins, and the pCREB (S133) level was also reduced, indicating that CREB degradation might be increased by KG-501 treatment. Wang et al. [32] reported that YAP may help stabilize CREB by inhibiting mitogen-activated protein kinase 14 (MAPK14/p38) and beta-transducin repeat containing E3 ubiquitin protein ligase (BTRC). Therefore, the decreased CREB level and pCREB (S133) in the OE+KG-501 group may be explained by the decreased YAP level induced by KG-501 treatment. However, the exact mechanism by which KG-501 treatment leads to decreased CREB levels in MSF cell lines requires further experimental exploration.

However, some limitations existed concerning the in vivo experiments. We only identified the RAMP1 expression of wound tissue protein during wound healing by Western blot analysis and IHC staining analysis, but not other molecules in the Gαi3-PKA-CREB-YAP axis signalling pathway. We can theoretically conclude that the axis was also the signalling pathway by which RAMP1 regulates wound healing because these pathway interventions significantly affected the early phase of wound healing and the expression level of RAMP1 gradually increased during the early phase of the wound healing process, which is also the proliferative phase where fibroblasts play a crucial role [3, 5]. However, we can only logically obtain the result that the intervention could change the wound healing process and that the corresponding molecule was important for wound healing. A complete understanding of the detailed confirmation of the mechanism awaits further studies that may require RAMP1 transgenic mice; however, animals are currently unavailable.

## Conclusions

In summary, our study identified, for the first time, that total RAMP1 levels change during skin wound healing and that RAMP1 OE alone can promote MSF proliferation by activating Gαi3 and downstream PKA and CREB, leading to *YAP* transcription and elevated nuclear YAP levels. The results of this study expand our knowledge of RAMP1 function and serve as the basis to exploit RAMP1 as a target in the treatment of skin wounds.

## Supplementary Information

**Additional file 1: Table S1.** Primer sequences used in lentivirus overexpression. **Table S2.** siRNA target sequences used for gene expression interference. **Table S3.** Primer sequences used for PCR amplification. **Table S4. **Predicted CBSs on the YAP promoter.

**Additional file 2: Fig. S1.** (**A**) Typical appearance of wounds on the DMSO (10 μl)-treated control side (Ctrl) and VP (100 nmol)-treated side (VP) at 0 hours (days 0), 24 hours (days 1), days 3, days 5 and days 7 post-injury. (**B**) Time course of the wound closure rates of the DMSO- and VP-treated sides. (**C**) Typical appearance of wounds on the siNC (5 nmol)-treated control side (siNC) and siGαi3-2 (5 nmol)-treated side (siGαi3-2) at 0 hours (days 0), 24 hours (days 1), days 3, days 5 and days 7 post-injury. (**D**) Time course of the wound closure rates of the siNC- and siGαi3-2-treated sides. (**E**) Typical appearance of wounds on the DMSO (10 μl)-treated control side (Ctrl) and H-89 (100 nmol)-treated side (H-89) at 0 hours (days 0), 24 hours (days 1), days 3, days 5 and days 7 post-injury. (**F**) Time course of the wound closure rates of the DMSO- and H-89-treated sides. (**G**) Typical appearance of wounds on the DMSO (10 μl)-treated control side (Ctrl) and BUC (100 nmol)-treated side (BUC) at 0 hours (days 0), 24 hours (days 1), days 3, days 5 and days 7 post-injury. (**H**) Time course of the wound closure rates of the DMSO- and BUC-treated sides. (**I**) Typical appearance of wounds on the DMSO (10 μl)-treated control side (Ctrl) and KG-501 (100 nmol)-treated side (KG-501) at 0 hours (days 0), 24 hours (days 1), days 3, days 5 and days 7 post-injury. (**J**) Time course of the wound closure rates of the DMSO- and KG-501-treated sides. The data are presented as means ± SD, and significant differences were evaluated using unpaired *t test*. *P < 0.05, **P < 0.01, ***P < 0.005

**Additional file 3: Fig. S2.** (**A**) CBSs in *YAP* promoter sequences analysed using the JASPAR database. (**B**) CUT&RUN assay and qPCR of CBSs on the YAP promoter. The experiments were performed in triplicate. The data are presented as means ± SD, and significant differences were evaluated using unpaired *t test*. *P < 0.05, **P < 0.01 and ***P < 0.005.

## Data Availability

All data generated or analysed during this study are included in this published article and its Additional information files.

## Abbreviations

CGRP: Calcitonin gene-related peptide
RAMP1: Receptor activity‐modifying protein 1
MSF: Mouse skin fibroblast
DOX: Doxycycline
qPCR: Quantitative real-time polymerase chain reaction
IF: Immunofluorescence
YAP: Yes-associated protein
G protein: Guanine nucleotide-binding protein
Gαi3: Inhibitory guanine nucleotide-binding protein (G protein) α subunit 3
PKA: Protein kinase A
cAMP: Cyclic adenosine monophosphate
CREB: Cyclic adenosine monophosphate (cAMP) response element-binding protein
CLR: Calcitonin receptor-like receptor
RCP: Receptor component protein
GPCR: G protein-coupled receptor
Gαs: Stimulatory Gα subunit
AC: Adenylyl cyclase
Gαi: Inhibitory Gα subunit
DMEM: Dulbecco’s modified Eagle’s medium
FBS: Foetal bovine serum
BUC: Bucladesine sodium
VP: Verteporfin
DMSO: Dimethyl sulfoxide
siRNAs: Small interfering RNAs
H&E: Hematoxylin–eosin
IHC: Immunohistochemical
DAB: Diaminobenzidine
IOD: Integrated optical density
PBS: Phosphate-buffered saline
HRP: Horseradish peroxidase
CUT&RUN: Cleavage under targets & release using nuclease
mAb: Monoclonal antibody
H3K4me3: Trimethylated histone H3 (Lys4)
CBS: CREB-binding site
SD: Standard deviation
VEC: Vector control
OE: Overexpression
CRE: CAMP responsive element
CBP: CREB-binding protein
MAPK14: Mitogen-activated protein kinase 14
BTRC: Beta-transducin repeat containing E3 ubiquitin protein ligase
LNF: Lane normalization factor
WT: Wild-type
Mut: Mutated
