# Supplementary material for: Receptor activity‐modifying protein 1 regulates mouse skin fibroblast proliferation via the Gαi3-PKA-CREB-YAP axis
Source: Cell Commun Signal. 2022 Apr 12;20:52. doi: 10.1186/s12964-022-00852-0 (PMC9004193; doi:10.1186/s12964-022-00852-0)
Supplement: Supplementary file 2 — Additional file 1: Table S1. Primer sequences used in lentivirus overexpression. Table S2. siRNA target sequences used for gene expression interference. Table S3. Primer sequences used for PCR amplification. Table S4. Predicted CBSs on the YAP promoter. [file 12964_2022_852_MOESM2_ESM.docx]

**Table S1.Primer sequences used in overexpression lentivirus.**

| **Gene** | **Primer sequences (5’ to 3’)** |
| --- | --- |
| **RAMP1-Forward** | **AACCGTCAGATCGCACCGGCGCCACCATGGCCCCGGGCCTGCGGGGCCTCCCG** |
| **RAMP1-Reverse** | **TCCTTGTAGTCCATGAATTCCACGATGCCCTCTGTGCGCTTGC** |

**Table S2. siRNA target sequences used for gene expression interference**

| **Gene** | **Target sequence (5’ to 3’)** |
| --- | --- |
| siGαi3-1 | **GGAATGTGGGCTTTATTGA** |
| siGαi3-2 | **GAAGATCTGAACCGAAGAA** |

**Table S3. Primer sequences used for PCR amplification**

| **Gene** | **Sequences or Target Sequences** **（5’-3’）** |
| --- | --- |
| **RAMP1 (Forward Sequence)**  **RAMP1 (Forward Sequence)**  **YAP1 (Forward Sequence)**  **YAP1 (Reverse Sequence)**  **Gαi3 (Forward Sequence)**  **Gαi3 (Reverse Sequence)**  **CREB(Site 1) (Forward Sequence)**  **CREB(Site 1)( (Reverse Sequence)**  **CREB(Site 2) (Forward Sequence)**  **CREB(Site 2)( (Reverse Sequence)**  **CREB(Site 3) (Forward Sequence)**  **CREB(Site 3)( (Reverse Sequence)**  **β-actin(Forward Sequence)**  **β-actin (Reverse Sequence)** | **CTGCTGGCTCACCATCTCTTC**  **ACCATAGCGTCTTCCCAATAGTC**  **AAGGCCATGCTTTCGAAC**  **ATGGCTTGCTCCCATCCATC**  **AGCAAGATGATCGACCGCAACT**  **CGTCCTCTGAATAGCCGTCCTC**  **AAGGTTCTAAAACGGTTGTGGC**  **AATTACATCCACCTCATGTCTCTTA**  **AAAGCACCAAACTGCTTAAGTG**  **GTACTTTTAGTGACCGGCCCTC**  **GTCTACCCTGCATTGTTTACCCTT**  **GCTCAAATAACAAATGTGGGGCA**  **CATCCGTAAAGACCTCTATGCCAAC**  **ATGGAGCCACCGATCCACA** |

**Table S4. Predicted CREB binding sites on YAP promoter**

| **Site** | **Start** | **End** | **Strand** | **Predicted sequence (5’ to 3’)** |
| --- | --- | --- | --- | --- |
| **Site1** | **818** | **825** | **+** | **TGACTTCA** |
| **Site2** | **2664** | **2671** | **+** | **TGCCGTCA** |
| **Site3** | **1204** | **1211** | **+** | **TGATGTTA** |
