# Supplementary figures and images for: Receptor activity‐modifying protein 1 regulates mouse skin fibroblast proliferation via the Gαi3-PKA-CREB-YAP axis
Source: Cell Commun Signal. 2022 Apr 12;20:52. doi: 10.1186/s12964-022-00852-0 (PMC9004193; doi:10.1186/s12964-022-00852-0)

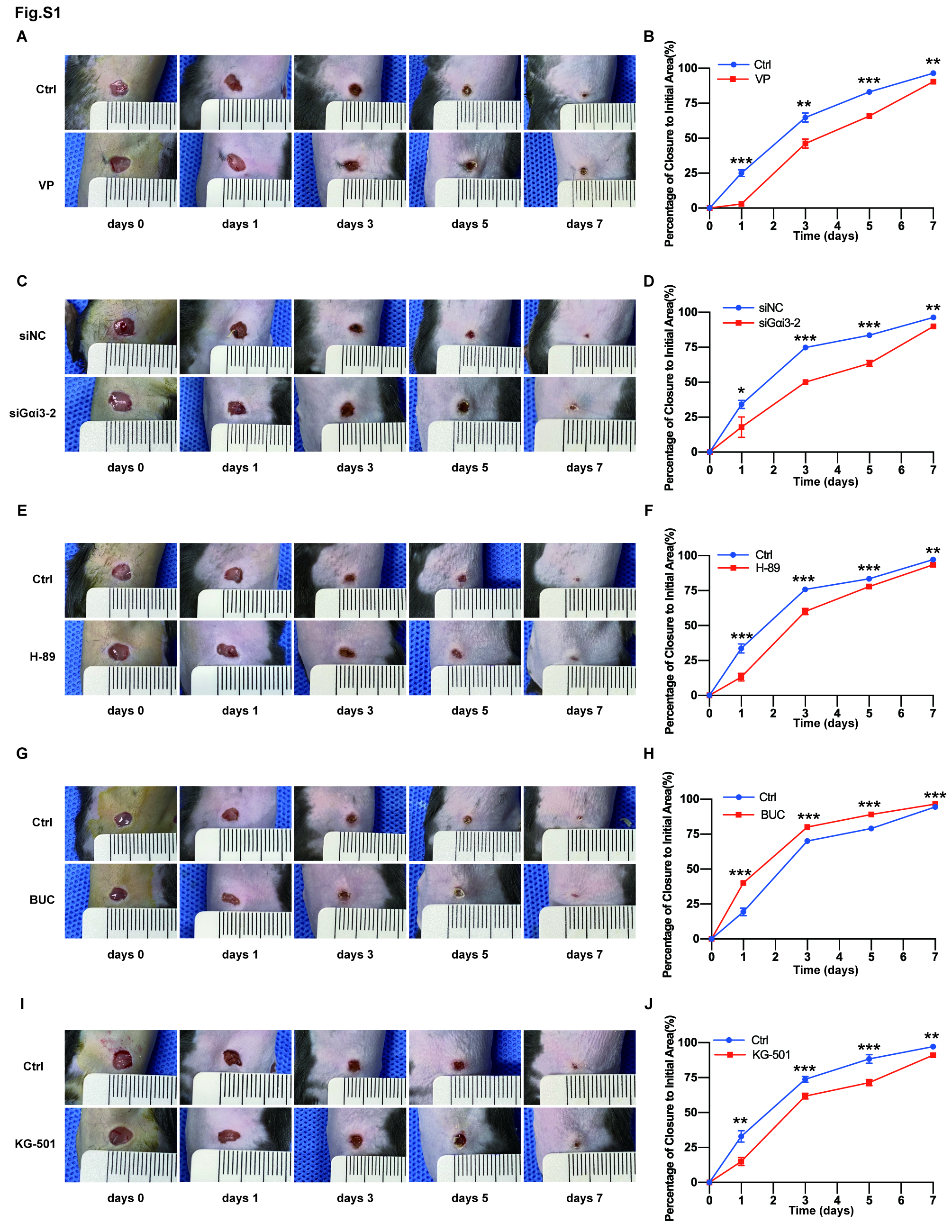

Supplement: Supplementary file 3 — Additional file 2: Fig. S1. (A) Typical appearance of wounds on the DMSO (10 μl)-treated control side (Ctrl) and VP (100 nmol)-treated side (VP) at 0 hours (days 0), 24 hours (days 1), days 3, days 5 and days 7 post-injury. (B) Time course of the wound closure rates of the DMSO- and VP-treated sides. (C) Typical appearance of wounds on the siNC (5 nmol)-treated control side (siNC) and siGαi3-2 (5 nmol)-treated side (siGαi3-2) at 0 hours (days 0), 24 hours (days 1), days 3, days 5 and days 7 post-injury. (D) Time course of the wound closure rates of the siNC- and siGαi3-2-treated sides. (E) Typical appearance of wounds on the DMSO (10 μl)-treated control side (Ctrl) and H-89 (100 nmol)-treated side (H-89) at 0 hours (days 0), 24 hours (days 1), days 3, days 5 and days 7 post-injury. (F) Time course of the wound closure rates of the DMSO- and H-89-treated sides. (G) Typical appearance of wounds on the DMSO (10 μl)-treated control side (Ctrl) and BUC (100 nmol)-treated side (BUC) at 0 hours (days 0), 24 hours (days 1), days 3, days 5 and days 7 post-injury. (H) Time course of the wound closure rates of the DMSO- and BUC-treated sides. (I) Typical appearance of wounds on the DMSO (10 μl)-treated control side (Ctrl) and KG-501 (100 nmol)-treated side (KG-501) at 0 hours (days 0), 24 hours (days 1), days 3, days 5 and days 7 post-injury. (J) Time course of the wound closure rates of the DMSO- and KG-501-treated sides. The data are presented as means ± SD, and significant differences were evaluated using unpaired t test. *P < 0.05, **P < 0.01, ***P < 0.005 [file 12964_2022_852_MOESM3_ESM.tif]

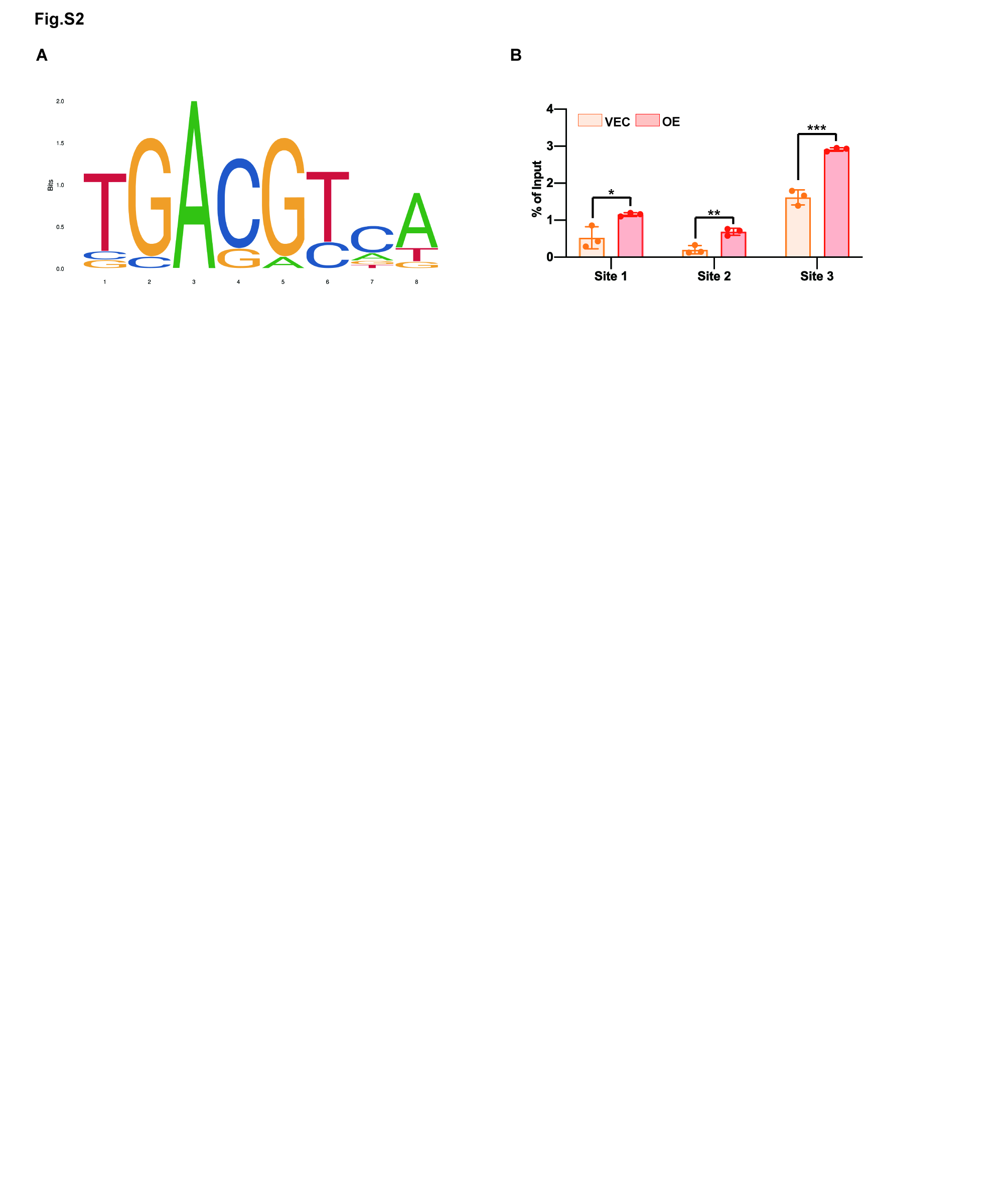

Supplement: Supplementary file 4 — Additional file 3: Fig. S2. (A) CBSs in YAP promoter sequences analysed using the JASPAR database. (B) CUT&RUN assay and qPCR of CBSs on the YAP promoter. The experiments were performed in triplicate. The data are presented as means ± SD, and significant differences were evaluated using unpaired t test. *P < 0.05, **P < 0.01 and ***P < 0.005. [file 12964_2022_852_MOESM4_ESM.tif]
